# Local control of sphincter‐preserving procedures and abdominoperineal resection for locally advanced low rectal cancer: Propensity score matched analysis

**DOI:** 10.1002/ags3.12032

**Published:** 2017-08-14

**Authors:** Ryosuke Okamura, Koya Hida, Tomohiro Yamaguchi, Tomonori Akagi, Tsuyoshi Konishi, Michio Yamamoto, Mitsuyoshi Ota, Shuichiro Matoba, Hiroyuki Bando, Saori Goto, Yoshiharu Sakai, Masahiko Watanabe, Japan Society of Laparoscopic Colorectal Surgery, Kazuteru Watanabe, Koki Otsuka, Ichiro Takemasa, Keitaro Tanaka, Masataka Ikeda, Chu Matsuda, Meiki Fukuda, Junichi Hasegawa, Shintaro Akamoto, Manabu Shiozawa, Atsushi Tsuruta, Takashi Akiyoshi, Takeshi Kato, Shunsuke Tsukamoto, Masaaki Ito, Masaki Naito, Akiyoshi Kanazawa, Takao Takahashi, Takashi Ueki, Yuri Hayashi, Satoshi Morita, Takashi Yamaguchi, Masayoshi Nakanishi, Hirotoshi Hasegawa, Ken Okamoto, Fuminori Teraishi, Yasuo Sumi, Jo Tashiro, Toshimasa Yatsuoka, Yoji Nishimura, Kenji Okita, Takaya Kobatake, Hisanaga Horie, Yasuyuki Miyakura, Hisashi Ro, Kunihiko Nagakari, Eiji Hidaka, Takehiro Umemoto, Hideaki Nishigori, Kohei Murata, Fuminori Wakayama, Ryoji Makizumi, Shoichi Fujii, Eiji Sunami, Hirotoshi Kobayashi, Ryosuke Nakagawa, Toshiyuki Enomoto, Shinobu Ohnuma, Jun Higashijima, Heita Ozawa, Keigo Ashida, Fumihiko Fujita, Keisuke Uehara, Satoshi Maruyama, Masato Ohyama, Seiichiro Yamamoto, Takao Hinoi, Masanori Yoshimitsu, Masazumi Okajima, Shu Tanimura, Masayasu Kawasaki, Yoshihito Ide, Shoichi Hazama, Jun Watanabe, Daisuke Inagaki, Akihiro Toyokawa

**Affiliations:** ^1^ Department of Surgery Kyoto University Kyoto Japan; ^2^ Division of Colon and Rectal Surgery Shizuoka Cancer Center Hospital Shizuoka Japan; ^3^ Department of Gastroenterological and Pediatric Surgery Oita University Faculty of Medicine Oita Japan; ^4^ Department of Gastroenterological Surgery Cancer Institute Hospital of the Japanese Foundation for Cancer Research Tokyo Japan; ^5^ Department of Data Science Institute for Advancement of Clinical and Translational Science Kyoto University Hospital Kyoto Japan; ^6^ Gastroenterological Center Yokohama City University Medical Center Kanagawa Japan; ^7^ Department of Gastroenterological Surgery Toranomon Hospital Tokyo Japan; ^8^ Department of Gastroenterological Surgery Ishikawa Prefectural Central Hospital Ishikawa Japan; ^9^ Department of Surgery Kitasato University School of Medicine Kanagawa Japan

**Keywords:** intersphincteric resection, local recurrence, rectal cancer, sphincter preservation

## Abstract

Sphincter‐preserving procedures (SPPs) for surgical treatment of low‐lying rectal tumors have advanced considerably. However, their oncological safety for locally advanced low rectal cancer compared with abdominoperineal resection (APR) is contentious. We retrospectively analyzed cohort data of 1500 consecutive patients who underwent elective resection for stage II‐III rectal cancer between 2010 and 2011. Patients with tumors 2‐5 cm from the anal verge and clinical stage T3‐4 were eligible. Primary outcome was 3‐year local recurrence rate, and confounding effects were minimized by propensity score matching. The study involved 794 patients (456 SPPs and 338 APR). Before matching, candidates for APR were more likely to have lower and advanced lesions, whereas SPPs were carried out more often following preoperative treatment, by laparoscopic approach, and at institutions with higher case volume. After matching, 398 patients (199 each for SPPs and APR) were included in the analysis sample. Postoperative morbidity was similar between the SPPs and APR groups (38% vs 39%; RR 0.98, 95% CI 0.77‐1.27). Margin involvement was present in eight patients in the SPPs group (one and seven at the distal and radial margins, respectively) and in 12 patients in the APR group. No difference in 3‐year local recurrence rate was noted between the two groups (11% vs 14%; HR 0.77, 95% CI 0.42‐1.41). In this observational study, comparability was ensured by adjusting for possible confounding factors. Our results suggest that SPPs and APR for locally advanced low rectal cancer have demonstrably equivalent oncological local control.

## 1 INTRODUCTION

Abdominoperineal resection (APR) has long been the standard operation for cancers located within 5 cm from the anal verge (AV). However, sphincter‐preserving procedures (SPPs) have increasingly been carried out in the last two decades and have recently been improved by more detailed anatomical understanding, improvements in surgical devices and techniques, accurate preoperative staging, and neoadjuvant therapy.^1^, ^2^ SPPs are now technically possible even for advanced or considerably low‐lying tumors. However, to determine whether this is, in fact, appropriate for advanced lesions, the oncological safety of these procedures must be confirmed.

Since Heald et al.^3^ reported excellent oncological results following SPPs with meticulous total mesorectal excision, many studies have oncologically compared SPPs with APR. A systematic review summarizing 24 studies published by 2010 concluded that rates of circumferential resection margin (CRM) involvement and local recurrence (LR) were significantly lower in SPPs than in APR.^4^ However, the authors also pointed out that, in these studies, tumors for APR were lower and more locally advanced. Distance from the AV is strongly associated with margin involvement and LR,^5^, ^6^, ^7^ and tumor stage should therefore be addressed. Candidates for APR also included patients with tumors that were ineligible for anastomotic procedures as a result of location or worse response to preoperative treatment. Another study showed that SPPs had better survival rates over APR after adjusting for age and tumor stage.^8^ However, there are no data regarding tumor height of each procedure group, and median distance from the AV in the whole study population was surprisingly 2 cm. In addition, open or laparoscopic approach, hospital caseload, and patients’ physical condition are associated with selection of sphincter preservation in clinical practice, as shown in several previous studies.^2^, ^9^, ^10^, ^11^ Moreover, many previous studies compared newer SPPs cases with older APR cases, or included many cases operated in the 1990s. The techniques and devices for rectal resection, preoperative accurate diagnosis, and preoperative treatment have advanced, especially in the last 10 years.^1^ Hence, we thought that the justification of oncological safety of SPPs would still be unclear and thus warranted a further well‐designed study to adjust for these confounders.

In the present study, we evaluated whether SPPs could achieve an adequate oncological clearance when confounders were adjusted. To accomplish this, we analyzed cohort data from recent cases and used propensity score matching to reduce confounding effects.

## 2 METHODS

### 2.1 Data source and study population

This study involves a secondary analysis of data obtained in our 2013 multicenter project that retrospectively evaluated the present status of surgical treatment for rectal cancer patients, with a comparison of laparoscopic surgery with open surgery designated as the primary analysis. The project was conducted by the Japan Society of Laparoscopic Colorectal Surgery in collaboration with 69 participating hospitals across Japan, and was registered in the UMIN Clinical Trials Registry (UMIN000013919). After approval of the protocol by the Central Institutional Review Board of the Japanese Society for Cancer of the Colon and Rectum and the institutional ethics committees of the participating institutions, we collected the demographic, clinicopathological, and first recurrence data for 1500 consecutive patients who underwent elective surgery for clinical stage II to III rectal cancer below the peritoneal reflection between January 2010 and December 2011 at 69 institutions. Median duration of follow up (interquartile range) was 3.5 years (2.9‐4.1). Rate of missing data on all variables of interest was extremely low (0.5%), owing to an adequately designed case report form and confirmatory data query.

For the present study, we analyzed data for patients with clinical T3‐4 Nany M0 tumor located 2‐5 cm from the AV. We excluded patients with such tumors located lower than 2 cm from the AV because SPPs were rarely carried out in such cases. Likewise, we excluded clinical T1‐2 patients because the proportion was also quite small and only a few patients underwent APR. Surgical procedures were either APR with permanent colostomy or SPPs including low anterior resection (LAR) and intersphincteric resection (ISR) with stapled or hand‐sewn coloanal anastomosis. Patients who underwent Hartmann procedure were excluded.

### 2.2 Data definitions

Incidence of LR was measured as the primary outcome. LR was defined as reappearance of a lesion located within the entire pelvic space diagnosed by imaging with or without biopsy; anastomotic, anterior space, presacral space, and lateral pelvic lymph node (LLN) recurrence were included in this definition.

The T stages were stratified into T3, T4a (penetrating to the level of the surface of visceral peritoneum), and T4b (invasive or adherent to other organs or structures).^12^ Stenosis was defined by inability to be traversed by the scope. Regional lymph nodes of rectal cancer included mesenteric lymph nodes along the inferior mesenteric or superior rectal arteries and LLN including the area of the obturator, internal iliac, external iliac, and common iliac.^13^ As a hospital characteristic affecting the clinical choice of SPPs and APR, we considered annual caseload,^9^, ^10^, ^14^ which was categorized into two groups using the median value: low volume (≤13 per year) and high volume (>13 per year) according to the number of annual surgical cases for advanced low rectal cancer.

### 2.3 Statistical analysis

Local recurrence rate was assessed by plotting Kaplan‐Meier curves, and Cox's proportional hazard model was used to estimate hazard ratios (HR) with 95% confidence intervals (CI). Dichotomous outcomes are expressed as relative risks (RR) with 95% CI. Continuous data and categorical data were compared with the Mann‐Whitney *U*‐test and Fisher's exact test, respectively, to assess statistical significance (*P*<0.05).

Propensity score is the probability that an individual patient would have been assigned to undergo an SPPs conditional on observed covariates.^15^, ^16^, ^17^, ^18^ The propensity score for each patient was generated using a logistic regression model based on factors potentially associated with the choice of procedure: specifically, patient factors (age, sex, body mass index [BMI], American Society of Anesthesiologists Physical Status [ASA‐PS], and comorbidity), tumor‐related factors (clinical T, clinical node involvement, tumor distance, and stenosis), and other factors (preoperative treatment, approach, and hospital caseload). Moreover, c‐statistic was calculated to confirm the discrimination accuracy of the multivariate model for score estimation. SPPs cases were matched 1:1 to APR cases with similar pre‐interventional probability (nearest‐neighbor matching with a maximum caliper width equal to [the standard deviation of the logit of propensity score]*0.2) without replacement.^19^ After matching, the covariates were considered balanced if the standardized differences were within ±0.1. Because this study was a secondary analysis of data obtained from our 2013 study, the sample size was mainly determined by the number of patients for whom data were available. All statistical analysis was done using JMP Pro version 12 software (SAS institute, Cary, NC, USA).

## 3 RESULTS

### 3.1 Patient characteristics

**Figure 1:**
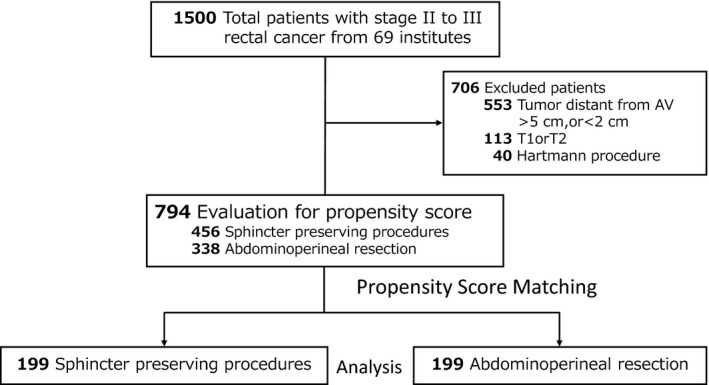
Study population. Flowchart of patient enrolment

We included 794 eligible patients from the original cohort: 456 (57%) underwent SPPs and 338 (43%) underwent APR (Figure 1). Propensity scores were estimated for each patient (Table 1), and the c‐statistic indicated high discrimination ability (82%). A total of 199 patients each for SPPs and APR were eventually included in the analysis sample.

**Table 1:** Propensity score calculation: Derived from 13 patient characteristics

Confounding factors	Coeff	SE	*P*	Odds ratio (95% CI)
Age, y (continuous)	−0.04	0.009	<0.001	0.96 (0.94‐0.98)
Male	−0.31	0.10	0.002	0.54 (0.36‐0.79)
BMI, n (%), kg/m^2^				
≥25	0.27	0.11	0.01	0.59 (0.38‐0.89)
18.5‐25, reference	‐	‐	‐	‐
<18.5	0.17	0.12	0.21	0.71 (0.41‐1.22)
ASA≥3	0.60	0.22	0.007	0.30 (0.12‐0.69)
Comorbidity	0.10	0.09	0.27	0.81 (0.57‐1.17)
Clinical T stage				
3	0.05	0.22	0.81	0.90 (0.37‐2.11)
4a, reference	‐	‐	‐	‐
4b	0.68	0.25	0.007	0.26 (0.09‐0.68)
Clinical N stage, positive	−0.05	0.10	0.61	0.91 (0.62‐1.33)
Clinical LLN involvement	0.23	0.14	0.10	0.63 (0.37‐1.08)
Tumor distance from AV, cm (continuous)	0.98	0.09	<0.001	2.66 (2.23‐3.19)
Stenosis	0.16	0.17	0.35	0.72 (0.37‐1.42)
Preoperative therapy	−0.24	0.10	0.02	1.60 (1.08‐2.39)
Laparoscopic approach	−0.16	0.10	0.10	1.38 (0.94‐2.03)
Hospital caseload ≥13/y	−0.29	0.09	0.001	1.78 (1.26‐2.53)

ASA‐PS, American Society of Anesthesiologists Physical Status; AV, anal verge; BMI, body mass index; Coeff, regression coefficient; LLN, lateral pelvic lymph nodes; SE, standard error.

Table 2 shows patient baseline characteristics before and after matching. In clinical practice, candidates for APR were likely to have poorer physical status, more lesions that were locoregionally advanced, and more lower‐lying lesions; SPPs were more often carried out following preoperative treatment, by laparoscopic surgery, and at institutions with high annual case volume. After the matching, better balance between the two groups could be observed for all variables. One‐third of the patients received preoperative treatment, and there were no significant differences between the two treatment groups in the clinical response rate defined by Response Evaluation Criteria In Solid Tumors^20^ (complete or partial response, 78% vs 77%, *P*=0.84).

**Table 2:** Clinical characteristics before and after propensity score matching

Characteristics	Before matching (n=794)		Standardized difference^a^	*P*	After matching (n=398)		Standardized difference	*P*
	SPPs (n=456)	APR (n=338)			SPPs (n=199)	APR (n=199)		
Age, median (range), y	62 (21‐89)	66 (31‐93)	0.44	<0.001	63 (21‐84)	64 (31‐87)	−0.07	0.55
Gender, n (%)								
Male	307 (67)	244 (72)	−0.11	0.14	149 (75)	145 (73)	0.05	0.73
Female	149 (33)	93 (28)	0.11		50 (25)	54 (27)	−0.05	
BMI, n (%), kg/m^2^								
<18.5	50 (11)	49 (15)	−0.12	0.02	20 (10)	21 (11)	−0.03	0.98
18.5‐25	321 (71)	206 (61)	−0.21		132 (66)	132 (66)	0.00	
≥25	84 (18)	81 (24)	−0.15		47 (24)	46 (23)	0.02	
ASA‐PS, n (%)								
1	194 (43)	99 (29)	0.29	<0.001	73 (37)	67 (34)	0.06	0.75
2	250 (55)	210 (63)	−0.16		119 (60)	123 (62)	−0.04	
3	9 (2)	27 (8)	−0.28		7 (3)	9 (5)	−0.10	
Comorbidity, n (%)	221 (49)	196 (58)	−0.18	0.01	102 (51)	106 (53)	−0.04	0.76
Diabetes mellitus	59 (13)	62 (18)			30 (15)	36 (18)		
Heart disease	22 (5)	23 (7)			11 (6)	13 (7)		
Cerebrovascular diseases	11 (2)	16 (5)			3 (2)	9 (5)		
Hypertension	104 (23)	84 (25)			48 (24)	45 (23)		
Others	88 (19)	95 (28)			40 (20)	51 (26)		
Clinical T stage, n (%)								
T3	406 (89)	260 (77)	0.32	<0.001	165 (83)	171 (86)	−0.08	0.58
T4a	21 (5)	11 (3)	−0.10		11 (6)	7 (4)	0.09	
T4b	29 (6)	66 (20)	−0.43		23 (12)	21 (11)	0.03	
Clinical N stage, n (%)								
Negative	295 (43)	140 (42)	0.02	0.72	81 (41)	85 (43)	−0.04	0.76
Positive	360 (57)	197 (58)	−0.02		118 (59)	114 (57)	0.04	
Clinical LLN involvement, n (%)	53 (12)	53 (16)	−0.12	0.11	28 (14)	24 (12)	0.06	0.66
Distance from AV, median (range), cm	4.0 (2.0‐5.0)	3.0 (2.0‐5.0)	0.91	<0.001	4.0 (2.0‐5.0)	4.0 (2.0‐5.0)	−0.03	0.77
Stenosis, n (%)	27 (6)	26 (11)	−0.18	0.02	13 (7)	16 (8)	−0.04	0.70
Preoperative treatment, n (%)	172 (38)	99 (29)	0.19	0.02	66 (33)	67 (34)	−0.02	>0.99
Chemoradiation	124 (27)	73 (22)			48 (24)	52 (26)		
Chemotherapy	37 (8)	21 (6)			17 (9)	12 (6)		
Radiation	11 (2)	5 (1)			1 (1)	3 (2)		
Approach, n (%)								
Open	270 (59)	234 (69)	0.21	0.005	131 (66)	133 (67)	−0.02	0.92
Laparoscopic	186 (41)	204 (31)	−0.21		68 (34)	66 (33)	0.02	
Hospital caseload, n (%)								
Low volume (<13/y)	183 (40)	191 (57)	−0.35	<0.001	109 (55)	100 (50)	0.10	0.37
High volume (≥13/y)	273 (60)	147 (43)	0.35		90 (45)	99 (50)	−0.10	
Procedure, n (%)								
LAR	290 (64)	‐		‐	111 (56)	‐		‐
ISR	166 (36)	‐			88 (44)	‐		

a Standardized difference is defined as the difference in means, scaled by the square root of the average of the two within‐group variances: $d=\left(\bar{x_{1}}-\bar{x_{2}}\right)/\sqrt{s_{1}^{2}+s_{2}^{2}/2}$, where $\bar{x_{1}}$, $\bar{x_{2}}$ are group means, and $s_{1}^{2},\;s_{2}^{2}$ are group variances.

ASA‐PS, American Society of Anesthesiologists Physical Status; AV, anal verge; BMI, body mass index; ISR, intersphincteric resection; LAR, low anterior resection; LLN, lateral pelvic lymph node.

### 3.2 Surgical and pathological findings

Simultaneous LLN dissection was carried out in 108 patients in the SPPs group and in 105 patients in the APR group (54% and 53%, *P*=0.84), and a diverting stoma was fashioned in 163 patients in the SPPs group (82%). Median intraoperative blood loss was lower in the SPPs group (Table 3). Intraoperative tumor perforation occurred in one patient who had ISR, and urethral injuries occurred in two patients (1 each for LAR and APR, respectively). A higher incidence was found for postoperative pelvic abscess and for wound infection in the APR group, whereas anastomotic leakage following SPPs occurred in 12% of cases. Overall morbidity was similar in both groups. Compared with the APR group, the SPPs group had a lower rate of blood transfusion, longer time to oral intake, and shorter hospital stay.

**Table 3:** Intra‐ and postoperative outcomes and pathological findings for matched sample of patients undergoing SPPs and APR

Variables	SPPs (n=199)	APR (n=199)	Relative risk (95% CI)	*P*
Operation time, median (IQR), m	340 (110‐798)	355 (141‐805)	‐	0.45
Intraoperative blood loss, median (IQR), mL	335 (10‐7040)	444 (10‐13500)	‐	0.02
Autonomic nerve preservation, n (%)	184 (93)	176 (90)	1.03 (0.98‐1.10)	0.27
Intraoperative adverse event, n (%)^a^	4 (2)	3 (2)	1.33 (0.30‐5.88)	>0.99
Morbidity, n (%), Grade II ≤^b^	76 (38)	77 (39)	0.98 (0.77‐1.27)	>0.99
Anastomotic leakage	24 (12)	‐	‐	‐
Pelvic abscess	8 (4)	18 (9)	0.44 (0.20‐1.00)	0.07
Wound infection	7 (7)	30 (15)	0.23 (0.10‐0.52)	<0.001
Urinary retention	15 (8)	11 (6)	1.36 (0.64‐2.89)	0.54
Ileus	18 (9)	17 (9)	1.06 (0.56‐1.99)	>0.99
Others	22 (11)	17 (9)	1.29 (0.71‐2.36)	0.50
Morbidity, n (%), Grade III ≤	35 (18)	37 (19)	0.95 (0.62‐1.44)	0.90
Reoperation	7 (4)	4 (2)	1.75 (0.52‐5.88)	0.54
Mortality	1 (1)	0 (0)	‐	‐
Blood transfusion, n (%)	29 (15)	48 (24)	0.60 (0.39‐0.92)	0.02
Time to oral intake, median (IQR), day	4 (3‐6)	3 (3‐4)	‐	0.04
Hospital stay, median (IQR), day	19 (13‐29)	22 (16‐32)	‐	0.004
Distal margin involvement, n (%)	1 (1)	‐	‐	‐
Radial margin involvement, n (%)	7 (4)	12 (6)	0.58 (0.23‐1.45)	0.35
Pathological T stage, n (%)				
T0/Tis/T1/T2	54 (27)	45 (23)	‐	0.14
T3	135 (68)	138 (69)		
T4a	2 (1)	0 (0)		
T4b	8 (4)	16 (8)		
Pathological N stage, n (%)				
Negative	117 (59)	111 (56)	‐	0.61
Positive	82 (41)	88 (44)		
Pathological LLN involvement, n (%)^c^	15/108 (14)	23/105 (22)	‐	0.15
Visible residual tumor	0 (0)	0 (0)	‐	‐
Adjuvant chemotherapy, n (%)	76 (39)	74 (37)	1.04 (0.81‐1.34)	0.84
First recurrence sites, n (%)^d^				
Local	20 (10)	23 (12)	0.87 (0.49‐1.53)	0.75
Liver	10 (5)	2 (6)	0.83 (0.37‐1.88)	0.83
Lung	17 (9)	28 (14)	0.61 (0.34‐1.07)	0.11
Peritoneum	1 (1)	1 (1)	1.00 (0.06‐15.9)	>0.99
Distant lymph node	4 (2)	7 (4)	0.57 (0.17‐1.92)	0.54
Others^e^	2 (1)	5 (3)	0.40 (0.08‐2.04)	0.45

a National Cancer Institute's Common Terminology Criteria for Adverse Events.

b Clavien‐Dindo Classification system.

c Only among the patients who underwent LLN dissection.

d Multiple sites were allowed.

e SPPs (1 bone, 1 unknown); APR (2 bone, 2 adrenal gland,1 brain).

APR, abdominoperineal resection; CI, confidence interval; IQR, interquartile range; LLN, lateral pelvic lymph node; SPPs, sphincter‐preserving procedure.

There were no significant differences in pathological T stage and nodal involvement between the treatment groups. Also, pathological tumor regression grade (TRG; the Japanese classification^21^) among patients who received preoperative treatment was comparable between the two groups: Grade 2‐3 (moderate to complete response), 38% and 42%; Grade 0‐1 (no regression to mild response), 62% and 58%, respectively (*P*=0.72). Rate of radial margin involvement was 4% and 6%, respectively, and only one patient in the SPPs group had distal margin (DM) involvement. However, out of 90 patients in the SPPs group who did not receive preoperative radiotherapy and for whom pathological DM length data were available, 20 (22%) had a DM<1 cm. Only 10 and 16 patients in the respective groups were pathological T4, and multivisceral resection of adjacent organs (uterus, vagina, seminal vesicle, prostate, urinary bladder, or coccyx) were consistently required in nine and 19 patients (5% and 10%).

### 3.3 Local recurrence

**Figure 2:**
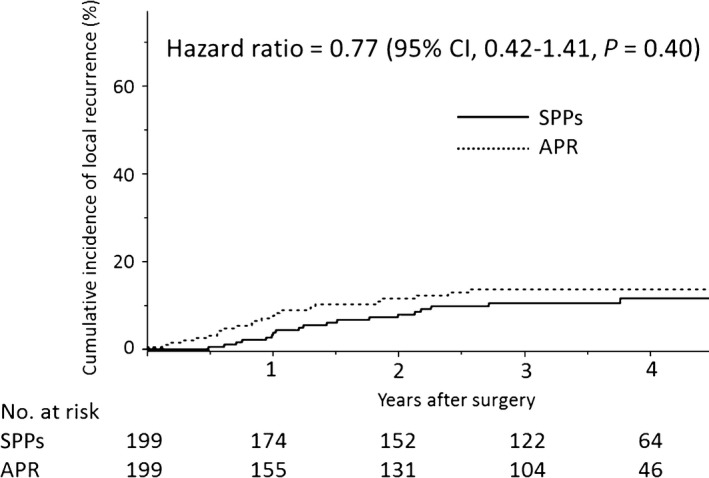
Cumulative incidence of local recurrence for matched sample of patients undergoing sphincter‐preserving procedures (SPPs) and abdominoperineal resection (APR)

**Figure 3:**
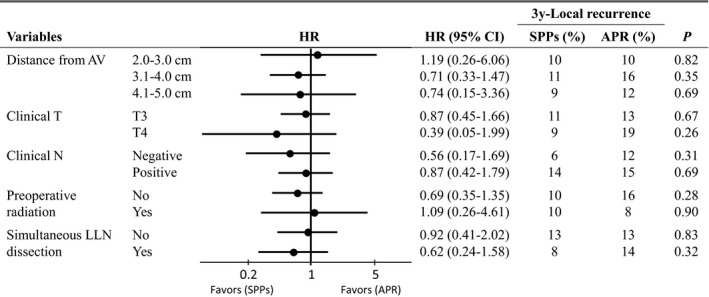
Subgroup analysis of local recurrence for matched sample of patients undergoing sphincter‐preserving procedures (SPPs) and abdominoperineal resection (APR). AV, anal verge; CI, confidence interval; HR, hazard ratio; LLN, lateral pelvic lymph node

Three‐year LR rate was 11% (95% CI 6.7‐16.1) in the SPPs group and 14% (95% CI 9.2‐19.8) in the APR group, and HR was 0.77 (95% CI 0.42‐1.41, *P*=0.40) (Figure 2). Stratification by subgroups revealed no large differences in the magnitude of the effect between the SPPs and APR groups (Figure 3). Among the 90 patients in the SPPs group who did not receive preoperative radiotherapy and whose pathological DM length was available, patients with negative DM <1 cm were likely to have a higher incidence of LR than those with DM ≥1 cm (25% vs 11%; RR 2.19, 95% CI 0.80‐5.95, *P*=0.15).

During the follow‐up period, 20 and 23 patients (10% vs 12%, *P*=0.75) experienced LR in the SPPs group and APR group, respectively. Salvage surgery for LR was more likely to be carried out in the SPPs group, although there was no significant difference (35% and 13%, *P*=0.15). Patterns of first recurrence in the two groups are also shown in Table 3. The stoma rate in the SPPs group at the time of the last follow up was 21% (41 of 199 patients).

## 4 DISCUSSION

Lack of randomized controlled trials (RCTs) makes it difficult to accurately compare surgical outcomes between SPPs and APR, and some surgeons remain concerned that SPPs may be disadvantaged on the issue of local control as a result of dissection or resection near the tumor and anastomosis. This study included a large number of patients with advanced low rectal cancer and demonstrated that SPPs had oncologically comparative local control to APR with several advantages in postoperative outcomes even after adjusting for confounders. Although many unmatched patients were excluded, we targeted only patients who were candidates for both strategies in order to ensure an accurate comparison and we used propensity score matched analysis to minimize confounding effects. In the end, we successfully matched APR patients and SPPs patients who had similar findings in pathological stage and TRG. Therefore, our study has higher comparability and probably more reliable results than previous reports on the same clinical question.

Local recurrence rate, the primary outcome of this study, was good in both SPPs and APR. The rates among only clinical T3 patients (11% and 13%, in Figure 3) were consistent with results of lower lesions in the randomized COLOR‐II trial (4%‐13%).^6^ Our data have some features such as simultaneous LLN dissection or lower rate of radiotherapy, but we surmise that our results would be generalizable to other countries because similar tendencies could be observed even after stratification by these factors (Figure 3). Regarding radial margin involvement rate, an established risk factor of LR,^22^, ^23^, ^24^, ^25^ the respective results (4% and 6%) were not inferior to the CRM involvement rates from recent RCTs (3%‐22%) although the radial margins as to criterion of 1 mm or 2 mm were not evaluated in the present study.^26^, ^27^, ^28^, ^29^ However, it should be noted that most hospitals participating in the present study were leading institutes in cancer care in Japan, such as university hospitals and medical centers. SPPs cases that preoperatively were expected to undergo complete resection at these institutions were included in the cohort, and appropriate treatment strategy and acquisition of safe surgical skills are required to achieve favorable resection for locally advanced low rectal cancer.

Negative DM are essential for local control in SPPs. The rate of DM involvement was also low in the matched SPPs patients (1%) and occurred in only three patients (0.7%) among 456 patients undergoing SPPs in the whole cohort. However, our results showed that the proportion of patients with negative DM <1 cm was not small (22%) among patients without preoperative radiotherapy and that they were likely to have a higher incidence of LR than those with margins ≥1 cm. In a meta‐analysis of 13 studies, Fitzgerald et al.^30^ reported that a DM greater than 1 cm was favored if radiotherapy was not used, although patients treated with radiotherapy could expect good local control even with DM less than 1 cm. In addition, the oncological local control of SPPs might be influenced by overweighting. A large cohort study by Meyerhardt et al.^31^ showed that increasing BMI was associated with a higher chance of LR, especially in men. Although we cannot discuss patients with severely elevated BMI (BMI >35 m^2^/kg), a not uncommon feature in Western countries, our data indicated that patients with BMI ≥25 who underwent SPPs had a higher LR rate compared to those with BMI <25 (17% vs 8%; RR 2.16, 95% CI 0.94‐4.96). We speculate that this may be as a result of difficulty in securing adequate DM. In fact, when limited to patients who did not receive preoperative radiotherapy in the matched SPPs sample, the patients with BMI ≥25 were likely to have higher incidence of negative DM <1 cm compared with those with BMI <25 (33% vs 18%; RR 1.83, 95% CI 0.85‐3.93). Consequently, more careful assessment for DM length is needed at the time of initial staging, and preoperative radiotherapy or APR is necessary when there is the possibility that a DM ≥1 cm cannot be achieved.

Data on outcomes during hospitalization might also be informative for patients and physicians. SPPs were associated with lower blood loss and a lower transfusion rate. In terms of the 3‐days shorter hospital stay observed in the SPPs group, we must take into account the fact that 82% of the patients had a temporary stoma and usually required readmission for reversal. Moreover, salvage surgery for LR was less frequent in the APR group. Likely reasons would be absence of anastomotic recurrence, and increased adhesions or anatomical violation as a result of lack of reconstruction might affect that. APR seemed to have a slight increase in lung metastasis. However, we think it premature to conclude that the surgical procedure for APR would have facilitated lung metastasis. There were subtle differences between the SPPs and APR groups in the completion rate of planned chemotherapy (86% and 77%, *P*=0.12) and perioperative use of irinotecan or oxaliplatin (37% and 31%, *P*=0.41) among patients who received perioperative systemic chemotherapy (preoperative chemoradiotherapy, chemotherapy alone, or adjuvant chemotherapy). These findings suggest that the intensity of chemotherapy was not completely the same between the two groups. It was difficult to ensure homogeneity of other patient physical factors such as tolerability compared with the confounders considered, and hematogenous metastasis might be influenced by these differences.

This study has several important limitations. First, postoperative anal function and quality of life (QOL) could not be discussed in this article. A systematic review addressing whether QOL following SPPs is superior to that following APR reported the findings as being controversial.^32^ Not a few patients undergoing SPPs would suffer from functional problems after stoma reversal such as fecal incontinence or urgency,^33^ and the final stoma rate in the SPPs group was actually not low (21%). Therefore, indication for sphincter preservation and improvement postoperative QOL are also important issues, and we are currently conducting a prospective study evaluating anal function, urine voiding and sexual function [UMIN 000011750]. Second, not all confounding effects were addressed. Generally, SPPs are not preferable for poorly differentiated tumors as a result of distal intramural spread.^34^ Although the proportion would be only a few percent of the cohort,^26^, ^34^ it is possible that lack of data might have led to the slight difference in recurrence rate. Furthermore, if a tumor directly invades the intersphincteric plane, sphincter excision and a permanent stoma are needed to achieve a clear pathological resection margin. Therefore, further study is needed to explore the indications for and contraindications to SPPs based on detailed information from preoperative magnetic resonance imaging, as recently reported by the MERCURY‐II study group.^7^ Third, the follow‐up duration was relatively short. Although we thought that LR would generally be identified within 3 years after surgery^35^, ^36^, ^37^ and some RCT were also designed to compare the OS, DFS, or LR at this time point,^6^, ^38^, ^39^ longer follow up is required to confirm these results. We plan to follow up this cohort until 5 years after surgery.

In conclusion, our study identified that SPPs and APR were comparable in oncological local control, even in locally advanced low rectal cancer. Also, SPPs had advantages during hospitalization and a higher rate of salvage surgery even though the incidence of permanent stoma was not low. These findings could better support preoperative decision‐making and patient counseling.

## ACKNOWLEDGEMENTS

The authors thank all members of the institutions participating in the “Open vs Laparoscopic Surgery for Advanced Low Rectal Cancer Patients” project: Kazuteru Watanabe (NTT Medical Center Tokyo); Koki Otsuka (Iwate Medical University); Ichiro Takemasa (Osaka University); Keitaro Tanaka (Osaka Medical College); Masataka Ikeda (Osaka National Hospital); Chu Matsuda (Osaka General Medical Center); Meiki Fukuda (Osaka Red Cross Hospital); Junichi Hasegawa (Osaka Rosai Hospital); Shintaro Akamoto (Kagawa University); Manabu Shiozawa (Kanagawa Cancer Center); Atsushi Tsuruta (Kawasaki Medical University); Takashi Akiyoshi (Cancer Institute Hospital, Japanese Foundation for Cancer Research); Takeshi Kato (Kansai Rosai Hospital); Shunsuke Tsukamoto (National Cancer Center Hospital); Masaaki Ito (National Cancer Center Hospital East); Masaki Naito (Kitasato University); Akiyoshi Kanazawa (Kitano Hospital); Takao Takahashi (Gifu University); Takashi Ueki (Kyushu University); Yuri Hayashi (Kyoto University); Satoshi Morita (Department of Data Science, Institute for Advancement of Clinical and Translational Science, Kyoto University Hospital); Takashi Yamaguchi (National Hospital Organization, Kyoto Medical Center); Masayoshi Nakanishi (Kyoto Prefectural University of Medicine); Hirotoshi Hasegawa (Keio University School of Medicine); Ken Okamoto (Kochi University); Fuminori Teraishi (Kochi Health Sciences Center); Yasuo Sumi (Kobe University); Jo Tashiro (Saitama Medical University International Medical Center); Toshimasa Yatsuoka, Yoji Nishimura (Saitama Cancer Center); Kenji Okita (Sapporo Medical University); Takaya Kobatake (Shikoku Cancer Center); Hisanaga Horie (Jichi Medical University Hospital); Yasuyuki Miyakura (Saitama Medical Center Jichi Medical University); Hisashi Ro (Juntendo University); Kunihiko Nagakari (Juntendo University Urayasu Hospital); Eiji Hidaka (Showa University Northern Yokohama Hospital); Takehiro Umemoto (Showa University Fujigaoka Hospital); Hideaki Nishigori (Shinko Hospital); Kohei Murata (Suita Municipal Hospital); Fuminori Wakayama (Seihoku Chuoh Hospital); Ryoji Makizumi (St. Marianna University); Shoichi Fujii (Teikyo University); Eiji Sunami (Tokyo University); Hirotoshi Kobayashi (Tokyo Medical and Dental University); Ryosuke Nakagawa (Tokyo Women's Medical University Hospital); Toshiyuki Enomoto (Toho University Ohashi Medical Center); Shinobu Ohnuma (Tohoku University); Jun Higashijima (Tokushima University); Heita Ozawa (Tochigi Cancer Center); Keigo Ashida (Tottori University); Fumihiko Fujita (Nagasaki University); Keisuke Uehara (Nagoya University); Satoshi Maruyama (Niigata Cancer Center Hospital); Masato Ohyama (Hyogo Cancer Center); Seiichiro Yamamoto (Hiratsuka Municipal Hospital); Takao Hinoi (Hiroshima University); Masanori Yoshimitsu (Hiroshima City Asa Citizens Hospital); Masazumi Okajima (Hiroshima Citizens Hospital); Shu Tanimura (Fukuoka University Hospital); Masayasu Kawasaki (Bell Land General Hospital); Yoshihito Ide (Yao Municipal Hospital); Shoichi Hazama (Yamaguchi University); Jun Watanabe (Yokosuka Kyosai Hospital); Daisuke Inagaki (Yokohama Minami Kyosai Hospital); Akihiro Toyokawa (Yodogawa Christian Hospital).

## DISCLOSURE

Conflict of Interest: Authors declare no conflicts of interest for this work.

Approval of the Research Protocol: The protocol of this project has been approved by the Central Institutional Review Board of the Japanese Society for Cancer of the Colon and Rectum and the institutional ethics committees of the participating institutions (Committee of Kyoto University, Approval no. R0033), and it conforms to the provisions of the Declaration of Helsinki.

Registry and the Registration No. of the Study: The project was registered in the UMIN Clinical Trials Registry (UMIN000013919).
